# First molecular detection of *Francisella tularensis* in turtle (*Testudo graeca*) and ticks (*Hyalomma aegyptium*) in Northwest of Iran

**DOI:** 10.1016/j.ijppaw.2023.11.005

**Published:** 2023-12-11

**Authors:** Amir Tukmechi, Abdulghaffar Ownagh, Ahmad Enferadi, Peyman Khademi

**Affiliations:** Department of Microbiology, Urmia University, Urmia, Iran

**Keywords:** Turtle, Blood, *Hyalomma aegyptium*, *Francisella*, Nested-PCR

## Abstract

*Francisella tularensis*, causative agent of tularemia, is a contagious zoonotic ailment. This study was aimed to molecularly detect *F. tularensis* in tortoise blood (n = 100) and ticks (n = 100) collected in the West Azerbaijan province, Iran suing a *16SrRNA* gene of the *Francisella* genus through employment of the Nested-PCR technique. The identified ticks were s *Hyalomma aegyptium* by morphological analysis. Seven percent (with a 95% CI: 3.5%–13.75%) of animal blood samples yielded positive results for the presence of the *Francisella*. Meanwhile, the *Francisella* was identified in tick samples at a rate of fifteen percent (15%) (with a 95% CI: 9%–23%). The samples containing positive results were specifically classified as *F. tularensis* subsp. *holarctica*. The samples were taken from ticks belonging to the *H. aegyptium* species that were gathered in Oshnavieh, southern part of West Azerbaijan province, Iran. This research was aimed to validate the existence of *F. tularensis* in ticks found within the West Azerbaijan province. Consequently, it is vital to acknowledge the potential of these ticks to transmit the bacteria to both livestock and humans through tick bites in this specific area.

## Highlights

• *Francisella tularensis* subsp. *holarctica* was detected in turtles and ticks in Iran.
• A proportion of 1.6% of the ticks found on the turtles were contaminated with *Francisella.*
• *Hyalomma aegyptium* was abundant tick that found on turtles.

## Graphical abstract

**Figure:**
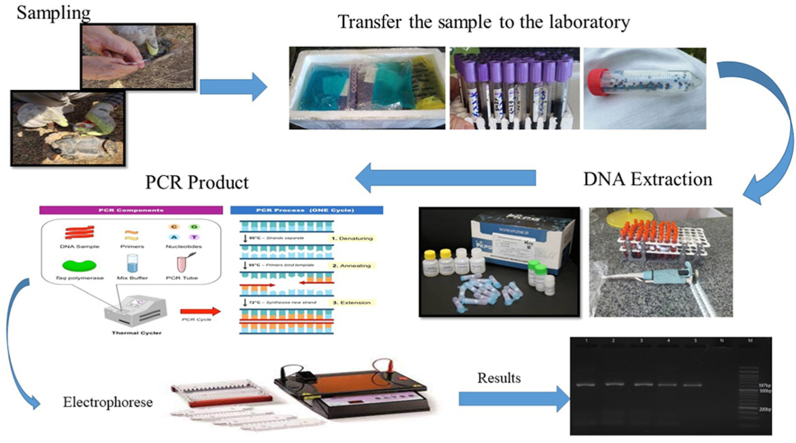

## 1 Introduction

*Francisella tularensis* functions is a zoonotic pathogen capable of inducing various clinical presentations of tularemia in humans. The range of clinical presentations that tularemia takes on depends on how the bacteria enter the body. Human transmission of *F. tularensis* occurs through routes encompassing skin and mucosal contact, the conjunctival pathway, as well as respiratory and gastrointestinal avenues. This microorganism is recognized to maintain its reservoir in a range of organisms, encompassing wild mammals, birds, fish, domesticated animals and arthropods (Sjöstedt, 2007).

Arthropods, particularly ticks, possess the ability to function as biological vectors for *F. tularensis*, potentially aiding in the spread of the infection to both humans and animals. The categorization of *F. tularensis* comprises three distinct subspecies (*tularensis*, *holarctica*, and *mediasiatica*), each exhibiting variations in reservoirs, life cycles and geographic dispersion. Among these, the tularensis and *holarctica* subspecies of *Francisella* are responsible for causing tularemia in humans. It is crucial to emphasize that *F. tularensis* subsp. tularensis is particularly noteworthy as the most potent subspecies in terms of its impact on both human and animal health (Sjöstedt, 2007).

It should be that *Francisella novicida*, which exists as a distinct species, is associated with uncommon illnesses mainly observed in individuals with weakened immune systems (Zellner and Huntley, 2019). *Francisella. tularensis* is predominantly encountered in the Northern Hemisphere, particularly in North America and Eurasia. Nevertheless, its existence has also been documented in Australia (Sjöstedt, 2007; Eden et al., 2017).

Signs of *F. tularensis* infection have surfaced across various regions of Iran, encompassing the western, northwestern, southwestern, northern, northeastern, and southeastern parts. This infection has been detected in human being as well as in wild mammals and livestock such as sheep, cows, hedgehogs, rodents and hares. In 1972, the initial documented report of contemporary *F. tularensis* infection in Iran described incidents involving 8 sheep, 3 cows and a hedgehog (Arata et al., 1973).

Ticks predominantly play a pivotal role in human and livestock tularemia, effectively sustaining the life cycle of *F. tularensis* over extended durations within the intermediary space connecting hosts and the environment (Sjöstedt, 2007). The occurrence of *F. tularensis* within ticks demonstrates global variation and has been documented in different tick genera and species, including *Dermacentor*, *Hyalomma*, *Haemaphysalis*, *Ixodes*, and *Rhipicephalus* (de Carvalho et al., 2016; Suzuki et al., 2016; Ghoneim et al., 2017; Koh et al., 2019).

Ticks facilitate bacterial transmission both through horizontal means such as saliva and feces as well as through vertical routes which encompass transovarial and transstadial transmission (Široký et al., 2010). *Hyalomma aegyptium* is a common tick on tortoises, lizards and snakes with a three-host life cycle. The main hosts are Palearctic tortoises of the genus *Testudo*, however, adults, nymphs and larvae target dogs, cattle, pigs, wild deer, jackals, wild boar, hares, foxes, mustelids, squirrels, hamsters, horses, hedgehogs, birds and humans (Apanaskevich, 2003; Girisgin et al., 2015; Akveran et al., 2020).

In a study by Akveran et al. (2020) in Turkey, the most prevalent pathogens identified molecularly in the ticks were *Hemolivia mauritanica* (51.9 %) followed by *Rickettsia aeschlimannii* (32.6 %), *Ehrlichia* spp. (30.2 %) and *Bartonella bovis* (0.8 %). All samples were negative for *Coxiella burnetii*, *Francisella tularensis*, *Anaplasma phagocytophilum*, *Babesia* spp., *Hepatozoon* spp. and *Theileria* spp.

The current study was planned to study *F. tularensis* in turtles **(***Testudo* *graeca*) (for the first time in Iran) and ticks **(***Hyalomma aegyptium*) from of West Azerbaijan Province, Iran.

## 2 Material and methods

### 2.1 Sample collection and study area

**Fig. 1:**
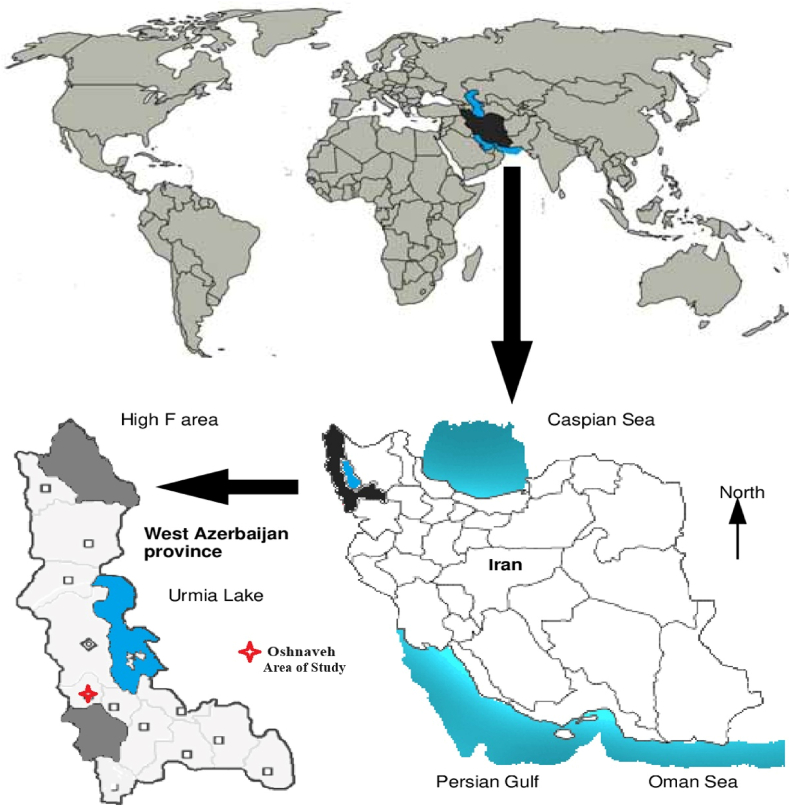
The schematic map of the studied areas, West Azerbaijan (Oshnavieh), Iran.

The present inquiry was carried out in the northwestern part of Iran, precisely within the West Azerbaijan province, covering the area of Oshnavieh (Fig. 1). The geographical coordinates of this location were documented as 36°91 N and 10°03 E. Throughout the spring and summer of 2022, an extensive gathering of 500 completely engorged adult ticks and 100 blood samples was carried out, involving a total of 100 spur-thighed tortoises (*Testudo graeca*). The ticks were carefully preserved in 70% ethanol and kept at a temperature of −20 °C until they were subjected to examination. All the ticks amassed during this effort were definitively identified as belonging to the *H. aegyptium* species.

To streamline the analysis procedure, the ticks were divided into 100 distinct samples, each comprising five ticks.

### 2.2 Sample collection

About 100 tick pool samples were gathered from various parts of land turtle in spring and summer in 2022 in northwest of Iran. The tick samples were put in sterile glass bottles with 4% methanol and 95% ethanol. The samples were then transferred to the parasitology laboratory of Faculty of Veterinary Medicine, Urmia University, for molecular tests and species identification. To identify adult ticks, a loupe microscope was used along with reliable diagnostic keys. Moreover, about 100 blood samples were selected randomly belonging to land turtles in West Azerbaijan. The blood samples were placed on ice and immediately transferred to the microbiology laboratory.

### 2.3 Molecular examination

#### 2.3.1 DNA extraction from turtle blood samples

DNA was extracted from the turtle blood samples utilizing the Wizard Genomic DNA purification kit (Addbio, Madison, USA), strictly adhering to the manufacturer's instructions. To initiate the process, a cautious transfer of 200 μl of whole blood was made into a 1.5 mL microtube. If the sample volume was less than 200 μl, Phosphate buffer saline (PBS) was incorporated to achieve a total volume of 200 μl. Subsequently, 20 μl of proteinase K was introduced into the microtube, succeeded by the addition of 200 μL of lysing buffer (FABG).

The microtube was vortexed and then positioned in a 60 °C Bain-Marie arrangement for 15 min. After a brief centrifugation, 200 μl of absolute ethanol was introduced to the sample.

#### 2.3.2 DNA extraction from tick's samples

DNA extraction from each tick homogenized sample pool was conducted using a column-based DNA extraction kit, precisely the Tissue and Blood 50 RXNS kit offered by Karmania Pars Gene Company (Iran). The extraction process was carried out in strict adherence to the manufacturer's instructions as outlined below: Initially, 30 gr of the complete sample pool was transferred into a 1.5 mL microtube. Following that, the sample was augmented with 250 μL of lysis buffer (LB) and vigorously vortexed for a duration of 5 s. Subsequently, the sample was allowed to incubate at room temperature for 5 min.

The resultant solution, which held the DNA, was carefully preserved at a temperature of −20 °C until it was ready for preparation in PCR analysis.

#### 2.3.3 Polymerase chain reaction

All samples were analyzed based on the presence of the *16S rRNA* gene through the utilization of specific primers employing the Nested-PCR technique (Forsman et al., 1994). Furthermore, the study encompassed the identification of the *F. tularensis* subspecies. This identification process involved assessing the *RD1* and *pdpD-2* genes using PCR subtyping methods, following the guidelines set forth by the WHO (2007). For the PCR, the second designed set of primers for *RD1*-F and *RD1*-R were assessed using Amplifix (Version 2.2.0) software.

Table 1 provides comprehensive information regarding the primer sequences and sizes of the gene products that were amplified to facilitate the identification of the subspecies. The PCR mixture was composed of 5 μL of the DNA sample, 10 μl of TEMpase Hot Start 2x Master Mix BLUE (sourced from Ampliqon, Denmark), and 900 nM concentrations for both the forward and reverse primers. This formulation resulted in a final volume of 25 μL, with the addition of deionized sterile distilled water.

**Table 1:** The PCR technique used for identifying the *Francisella* genus and subspecies includes specific primers, product sizes and a thermal program, which are described as follows.

Protocol	Primer Name	Sequence 5'----3'	product size (bp)		PCR condition (cycle)
Normal-PCR	16SrRNA-F	TGGTGTAGCGGTGAAATGCGTA	495		95 °C for 4 m, 95 °C for 55s, Touchdown 66–63 (5) for 55s, 72 °C for 55 m, 72 °C for 7 m. (32)
	16SrRNA-R	GCCTTGTCAGCGGCAGTCTTAATA			
Nested-PCR	16SrRNA-NF	TGGTAGTCCACGCTGTAAACGATG	306		95 °C for 4 m, 95 °C for 15s, 66 °C for 15s, 72 °C for 7 m. (30)
	16SrRNA-NR	GCGGGACTTAACCCAACATTTCAC			
Normal-PCR	*pdpD*-F	TGGGTTATTCAATGGCTCAG	*F. tularensis*	136	95 °C for 4 m, 95 °C for 15s, 66 °C for 15s, 72 °C for 7 m. (30)
			*F. holarctica*	–	
	*pdpD-R*	TCTTGCACAGCTCCAAGAGT	*F. mediasiatica*	280	
			*F. novicida*		
Normal-PCR	*RD1-F*	TGATTCTGTACGCTGGCGTTGT	*F. holarctica*	520	95 °C for 4 m, 95 °C for 15s, 62 °C for 15s, 72 °C for 7 m. (30)
	*RD1-R*	AAGCGCACCGTAGCTTTCATCT			

#### 2.3.4 Sequencing

The PCR products of four *Francisella* isolates, with the amplified fragment of *16S rRNA* gene (306 bp), and *RD1* gene (520 bp) for four samples, were sent to Pishgam Company (Tehran, Iran) for sequencing. Obtained nucleotide sequences were searched against GenBank (National Centre for Biotechnology Information, Rockville Pike, and Bethesda, USA) using the advanced BLAST similarity search option and compared to the same sequences of *Francisella* isolates from GenBank.

## 3 Results

### 3.1 Results based on *16S rRNA* gene

The findings revealed that among the 100 tick pool samples, a total number of 15 samples exhibited infection from the *Francisella*, identified through the presence of the *16S rRNA* gene (15%; 95% CI: 9%–23%). Additionally, within the turtle blood samples, the assessment demonstrated a contamination rate of seven out of 100 samples for the presence of the *Francisella* genus via the *16S rRNA* gene (7%; 95% CI: 3.5%–13.75%).

### 3.2 Detection of *Francisella tularensis* subspecies

The presence of *F. tularensis* was not detected through the *pdpD-2* gene within both blood and tick samples. However, the *RD1* gene did yield amplification, producing a 520 bp product size. In tick samples, the outcomes based on the *RD1* gene indicated the presence of 15 samples belonging to the *holarctica*. Similarly, from blood samples, seven instances were isolated that also fell under the *holarctica*.

### 3.3 Phylogenetic analysis

**Fig. 2:**
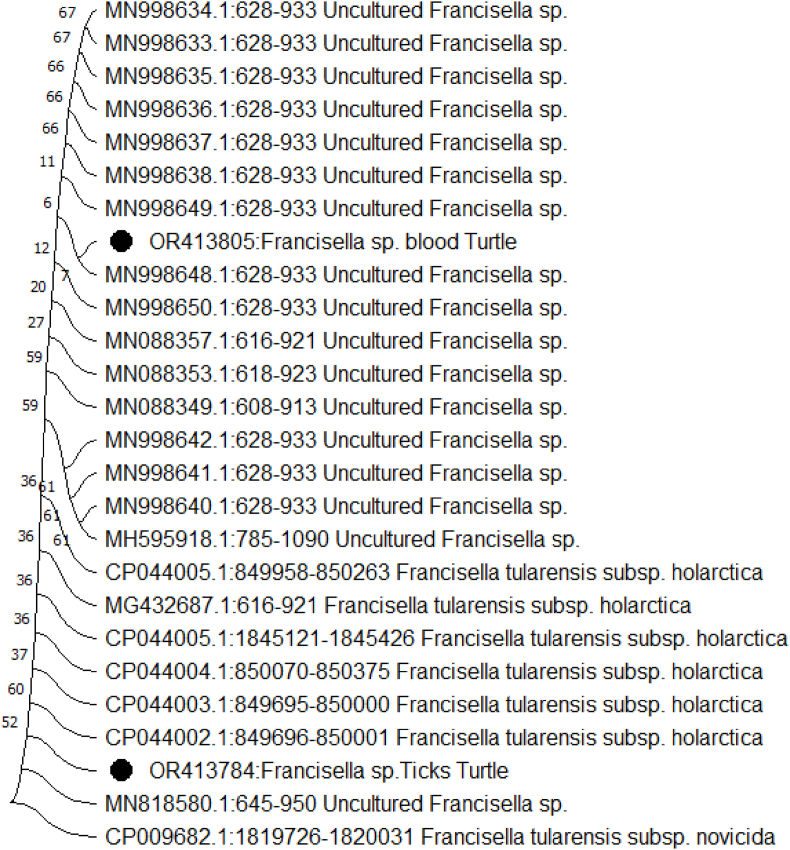
The evolutionary lineage was determined using the Maximum Likelihood method and the Tamura-Nei model. The displayed tree represents the one with the most favorable log likelihood (−429.22). Additionally, the branches are accompanied by the percentage denoting how frequently the related taxa formed clusters in the trees. The initial trees for exploratory purposes were automatically created using the Neighbor-Join and BioNJ algorithms. This was accomplished by utilizing a matrix of pairwise distances, which were calculated employing the Tamura-Nei model. From these initial trees, the one with the most favorable log likelihood value was selected. This analysis was conducted on a collection of 31 nucleotide sequences. The encompassed codon positions consisted of 1st+2nd+3rd + Noncoding. The final dataset consisted of a total of 306 positions. The evolutionary analyses were performed utilizing MEGA11.

**Fig. 3:**
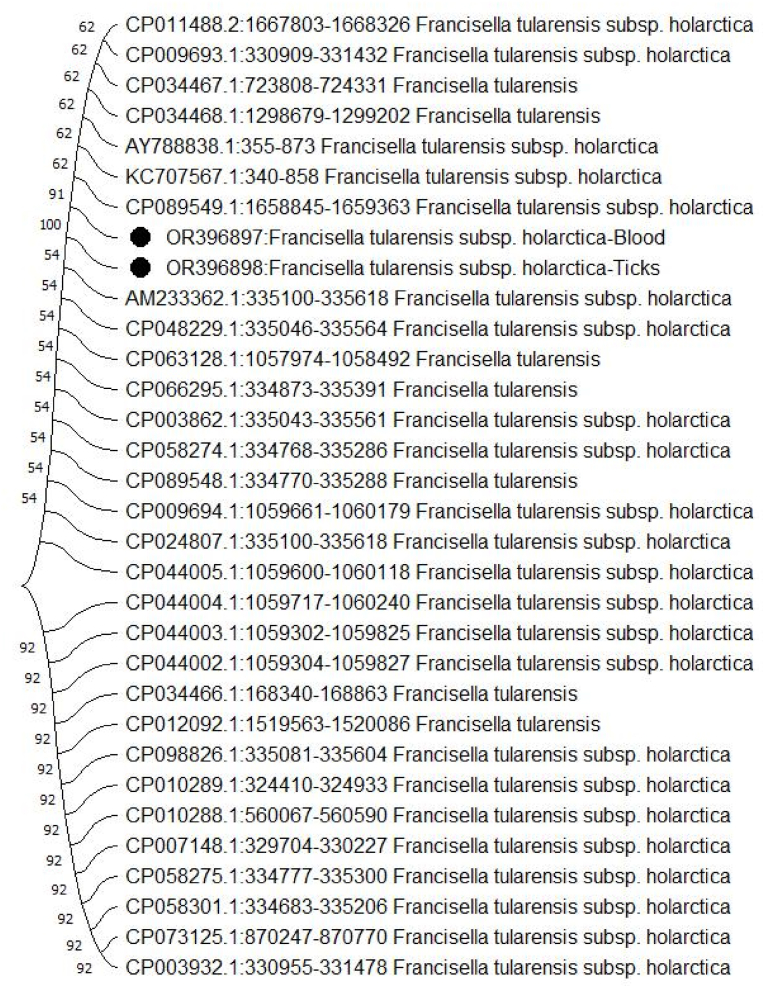
The lineage's evolutionary narrative was deduced through the application of the Neighbor-Joining technique. The most advantageous tree configuration is depicted. Adjacent to the branches, the percentages reflect how often the related taxa aggregated within the bootstrap test, comprising 1000 replicates. Evolutionary distances were calculated using the Maximum Composite Likelihood method, expressed as the count of base substitutions per site. In this study, a collective of 32 nucleotide sequences were taken into account. The codon positions covered 1st+2nd+3rd + Noncoding. Ambiguous positions were excluded for each sequence pair, following the pairwise deletion technique. In the culminating dataset, a collective count of 542 positions was encompassed. The evolutionary analyses were executed using MEGA11.

Through the construction of a phylogenetic tree utilizing neighbor-joining analysis of both the *16S rRNA* and *RD1* partial genes, it was discerned that four isolates exhibited close clustering. These isolates showcased a high degree of similarity, ranging between 99.9% and 100%, signifying their similarity as practically identical (Fig. 2, Fig. 3).

Fig. 2, Fig. 3 illustrate the outcomes of phylogenetic analyses carried out utilizing *RD1* and *16S rRNA* sequences. The nucleotide sequences recorded in this study have been assigned accession numbers (*16S rRNA*: OR413784, OR413805) and (*RD1*: OR396897, OR396898). These sequences have been added to the GenBank database, which can be accessed at https://www.ncbi.nlm.nih.gov/genbank/.

## 4 Discussion

The present study was the first study on *Francisella* in the blood samples and tick pool of West Azerbaijan province (Oshnavieh). Results of the present study revealed that 23% of the all examined tick samples were positive for *Francisella*. Also, the contamination of *Francisella* genus in blood samples was 7%.

Ticks play a significant role in transporting critical pathogens for both humans and animals, making them indicators of infection within natural ecosystems (Rizzoli et al., 2011). In recent times, there has been an expansion in the geographical range and habitats of diverse and adaptable tick species. This growth trend is substantially influenced by factors such as alterations in land utilization, shifts in climate patterns, and the global interconnectedness (Gubler et al., 2001; Harrus and Baneth, 2005). On the flip side, specific tick species, like *H. aegyptium*, demonstrate a pattern of shrinking geographical distribution, closely linked to the diminishing populations of their vulnerable hosts (Mihalca et al., 2011).

Nevertheless, as a broader principle, the decline in the presence of natural host populations may instigate a phenomenon known as host-switching behavior (Keesing et al., 2010). Given that *H. aegyptium* is known to shift its feeding preference towards a variety of other hosts, particularly during its pre-adult stages, the assessment of its zoonotic pathogen load holds significant importance.

In terms of their contribution to the ecology of zoonotic infectious diseases, tortoises and their ticks have received considerably less focus in comparison to mammals and birds. Among small mammals, hedgehogs (*Erinaceus* spp.) hold particular importance in synanthropic environments. In such contexts, they act as reservoir hosts for important human pathogens, including *A. phagocytophilum*, *Babesia* spp. (Silaghi et al., 2012), and *B. burgdorferi* s.l. (Skuballa et al., 2012). Considering *H. aegyptium*'s occasional feeding on hedgehogs and its potential interaction with humans (Bursali et al., 2010), it becomes essential to evaluate the role of this species as a carrier host for zoonotic pathogens.

In this study, the Real Time-PCR technique was employed to examine the presence of *F. tularensis* in both small ruminants and the associated ticks in the western region of Iran. Through this method, *F. tularensis* DNA was identified in 0.82% of tick samples collected from the Kurdistan province. However, no presence of the bacteria was observed in the sheep and goat populations (Rahravani et al., 2022).

Starting from 2011, the Pasteur Institute of Iran has shown growing interest in exploring tularemia within the country. Most of the research efforts in Iran have predominantly concentrated on rodents, the environment, and the human populations vulnerable to the disease. These studies have sounded cautionary notes about the potential emergence of tularemia outbreaks in the country.

Despite these previous warnings, there remains a substantial dearth of research conducted on *F. tularensis* in arthropod vectors within the Iranian context. Hence, a vital goal is to comprehend the origins of infection within the lifecycle of *Francisella* in Iran. As a part of this ongoing investigation, the DNAs of *F. tularensis* found in tick samples underwent molecular subtyping assays, leading to their categorization as *F. tularensis* subsp. This particular subspecies, *holarctica*, exhibits reduced virulence in both humans and animals and is widely distributed across Asia, Europe, and Eurasia (Sjöstedt, 2007; Rahravani et al., 2022). Research conducted in Turkey, a neighboring country to the northwest, has emphasized the existence of *F. tularensis* subsp. *holarctica* within its territory (Yeşilyurt et al., 2011; Duzlu et al., 2016).

This particular subspecies of *F. tularensis* is frequently linked to water sources that have been contaminated, such as lakes, rivers, and ponds. A previous investigation also proposed waterborne transmission as a potential avenue for a tularemia outbreak in Iran (Esmaeili et al., 2021).

Taking into account this collection of evidence along with the findings of the present study, there is a potential indication that subsp. *holarctica* is present within Iran. Given the infrequent occurrence of *F. tularensis* bacteremia (Haristoy et al., 2003), the task of isolating *F. tularensis* from blood samples of livestock presents a considerable challenge. The intricacies of this lifecycle could plausibly contribute to the difficulties encountered in successfully identifying *F. tularensis* within livestock blood samples. Past seroepidemiological investigations carried out in this area revealed that 16% of butchers had displayed positive results for tularemia antibodies (Esmaeili et al., 2014).

Moreover, instances of infection were documented among captured rodents (4.8%) and hunters (18%) within the Kurdistan province (Mostafavi et al., 2017). Hence, it strongly advocates for the initiation of comprehensive research endeavors on F. tularensis involving diverse tick species and other arthropods. Such investigations are vital to ascertain their plausible contribution to the epidemiological cycle of *F. tularensis* in Iran, particularly within regions prone to endemicity. A molecular study conducted in Egypt unveiled the presence of *Francisella* spp. among 4.7% of ticks found on camels.

Nevertheless, aligning with our findings, *Francisella* spp. was not identified in the blood and fecal samples of camels. Intriguingly, their study unveiled a remarkable seroprevalence of *F. tularensis* among individuals working in slaughterhouses, indicating a greater prevalence of tick bites (20.7%) in comparison to sporadic exposure (2.2%) among workers who encountered ticks less frequently (Ghoneim et al., 2017). Conversely, reinforcing the disparities observed, findings opposing these results were found in a study conducted in Malaysia.

The absence of *Francisella* spp. in ticks and animal samples collected from livestock farms suggests that its presence might be restricted to Dermacentor questing ticks, without spreading to different tick species and livestock animals. The study highlighted that 11.3% of questing ticks gathered from forest reserves exhibited positive results for the *16S rRNA* of *Francisella* spp. In stark contrast, no ticks attached to livestock animals such as sheep, goats, and cattle, nor their corresponding blood samples, displayed any indication of *Francisella* spp. presence (Koh et al., 2019).

Even though *F. tularensis* remains prevalent among human populations in Turkey (Akalın et al., 2009), employing the same method has not revealed its presence in different tick and mosquito species (Haristoy et al., 2003; Akalın et al., 2009; Duzlu et al., 2016; Demir et al., 2020). Consequently, it has been postulated that the pattern of tularemia outbreaks in Turkey might be driven by water-borne transmission rather than vector-borne transmission (Duzlu et al., 2016). In a Japanese study, *F. tularensis* demonstrated prevalence within *Ixodes monospinosus* ticks (8.22%), yet its occurrence in *Ixodes persulcatus* ticks was significantly lower at 0.66% (Suzuki et al., 2016). In this study, out of the 244 ticks collected from sheep and goats, the majority were identified as *D. marginatus* (66.4%), with *Rh. sanguineus* accounting for the minority (10%). The presence of *F. tularensis* DNA was exclusively detected in *D. marginatus* ticks (1.22%). These findings indicate a potential involvement of *D. marginatus* in the lifecycle of *F. tularensis* in the Kurdistan region.

Notably, in the United States, *Dermacentor variabilis* (*D. variabilis*) assumes a pivotal role as a vector for *F. tularensis*, actively participating in the bacterium's natural life cycle (Whitten et al., 2019; Zellner and Huntley, 2019). Whitten et al. have documented a tularemia prevalence of 34% in D. variabilis through a comprehensive RT-PCR study (Whitten et al., 2019). Similar to our study, previous research conducted in the USA and Europe has typically reported *F. tularensis* presence in various Dermacentor species ranging from less than 1% to less than 10% (Goethert et al., 2004; Bielawska-Drozd et al., 2018; Hubalek and Rudolf, 2017).

Employing a real-time TaqMan PCR assay targeting the *tul4* and *ISFtu2* genes, an examination was conducted on ticks acquired from both wild and domestic animals across the Iberian Peninsula. The research revealed the presence of *F. tularensis* in 0.6% of ticks. Notably, it's important to mention that positive instances were primarily linked to *Rh. sanguineus* (25.7%) and *D. marginatus* (2.4%) (de Carvalho et al., 2016).

Instances of *Francisella* and *Francisella*-like endosymbionts (FLEs) have been documented in *Rh. sanguineus* ticks in diverse locations, including Bulgaria (Ivanov et al., 2011), Romania (Andersson et al., 2018), and Thailand (Rakthong et al., 2016).

Given the insights from previous studies, our results suggest that the prevalence of *F. tularensis* in ticks could vary depending on the specific tick species. For a thorough understanding of the disease dynamics, encompassing reservoirs and the primary agents of infection transmission in areas where positive cases have been detected, it is recommended to conduct a study with a larger sample size.

## 5 Conclusions

During the current research, the blood of examined turtles and ticks collected from various regions in Iran were positive for *F. tularensis* bacteremia through the PCR and Nested-PCR techniques. Importantly, a proportion of 1.6% of the ticks found on these turtles (*H. aegyptium* ticks) were determined to harbor *F. tularensis* subsp. *holarctica*. Hence, it is advisable to evaluate the contribution of *H. aegyptium* ticks to the epidemiological cycle and the persistence of *F. tularensis* in Iran. Conducting more extensive screenings of both livestock and their associated ticks, encompassing a substantial volume of samples from diverse geographical regions throughout Iran is of great importance.

## Authors’ contributions

A.T., and A.O., conceptualized the study. A.E., P.K. and A.O Formal analysis. P.K, A.O, A.E and A.T., wrote the first draft of the article. All authors revised the article and approved the final version. A.T., and P.K., had full access to all of the data in the study and take responsibility for the integrity of the data and the accuracy of the data analysis.

## Funding

No fund.

## Data availability statement

The data that support the findings of this study are available upon reasonable request.

## Ethical statement

All procedures in this study were conducted in accordance with the Veterinary Ethics Committee of the Faculty of Veterinary Medicine of Urmia University (IRUU-AEC-165/AF2/January 04, 2021) and approved all protocols used in the present study.

## Declaration of competing interest

The authors declare no conflict of interest.
